# Dimethylethanolamine Decreases Epileptiform Activity in Acute Human Hippocampal Slices *in vitro*

**DOI:** 10.3389/fnmol.2019.00209

**Published:** 2019-09-06

**Authors:** Larissa Kraus, Florian Hetsch, Ulf C. Schneider, Helena Radbruch, Martin Holtkamp, Jochen C. Meier, Pawel Fidzinski

**Affiliations:** ^1^Charité-Universitätsmedizin Berlin, corporate member of Freie Universität Berlin, Humboldt-Universität zu Berlin, and Berlin Institute of Health, Department of Neurology with Experimental Neurology, Berlin, Germany; ^2^Berlin Institute of Health (BIH), Zoologisches Institut, Technische Universität Braunschweig, Braunschweig, Germany; ^3^Zoologisches Institut, Technische Universität Braunschweig, Braunschweig, Germany; ^4^Charité-Universitätsmedizin Berlin, corporate member of Freie Universität Berlin, Humboldt-Universität zu Berlin, and Berlin Institute of Health, Department of Neurosurgery, Berlin, Germany; ^5^Charité-Universitätsmedizin Berlin, corporate member of Freie Universität Berlin, Humboldt-Universität zu Berlin, and Berlin Institute of Health, Department of Neuropathology, Berlin, Germany

**Keywords:** epilepsy, drug development, human, hippocampus, *ex vivo*

## Abstract

Temporal lobe epilepsy (TLE) is the most common form of focal epilepsy with about 30% of patients developing pharmacoresistance. These patients continue to suffer from seizures despite polytherapy with antiepileptic drugs (AEDs) and have an increased risk for premature death, thus requiring further efforts for the development of new antiepileptic therapies. The molecule dimethylethanolamine (DMEA) has been tested as a potential treatment in various neurological diseases, albeit the functional mechanism of action was never fully understood. In this study, we investigated the effects of DMEA on neuronal activity in single-cell recordings of primary neuronal cultures. DMEA decreased the frequency of spontaneous synaptic events in a concentration-dependent manner with no apparent effect on resting membrane potential (RMP) or action potential (AP) threshold. We further tested whether DMEA can exert antiepileptic effects in human brain tissue *ex vivo*. We analyzed the effect of DMEA on epileptiform activity in the CA1 region of the resected hippocampus of TLE patients *in vitro* by recording extracellular field potentials in the pyramidal cell layer. Epileptiform burst activity in resected hippocampal tissue from TLE patients remained stable over several hours and was pharmacologically suppressed by lacosamide, demonstrating the applicability of our platform to test antiepileptic efficacy. Similar to lacosamide, DMEA also suppressed epileptiform activity in the majority of samples, albeit with variable interindividual effects. In conclusion, DMEA might present a new approach for treatment in pharmacoresistant TLE and further studies will be required to identify its exact mechanism of action and the involved molecular targets.

## Introduction

Epilepsy is a major neurological disorder affecting up to 65 million people worldwide (Hirtz et al., 2007; Ngugi et al., 2010). The need for adequate treatment is not only given by seizures itself along with associated risks of injury and premature death but also by comorbidities and social stigmatization. Specifically in focal epilepsy, 30%–40% of patients do not respond to currently available antiepileptic drugs (AEDs), resulting in pharmacoresistance with ongoing seizures despite treatments with multiple AEDs at high dosages (Stephen et al., 2001). Alternative therapies such as ketogenic diet or brain stimulation have been suggested to reduce seizure burden in pharmacoresistant patients (Giordano et al., 2014; Kowski et al., 2015; Dibué-Adjei et al., 2019). However, ketogenic diet has been shown to be effective in children and with modification in adults but is still rarely considered as treatment in adults (Hallböök et al., 2015; Falco-Walter et al., 2019). Ongoing investigations show promising seizure reduction in pharmacoresistant patients by deep brain stimulation (Zangiabadi et al., 2019). However, this approach requires optimal selection of targeted brain regions and prospective trials are lacking. Finally, surgical removal of the epileptic focus remains often the only treatment option for pharmacoresistant patients (Wiebe et al., 2001; Engel et al., 2007). Yet, only in a minority of patients, epilepsy is amenable to surgery, and only 60%–70% of resected patients have a positive outcome with substantial reduction of the seizure burden (International League Against Epilepsy Outcome Scale 1–2; Mohan et al., 2018). Thus, identification of new antiepileptic treatment options in focal pharmacoresistant epilepsy is of paramount importance.

Dimethylethanolamine (DMEA) has previously been investigated as a stimulant and treatment for several neurological diseases, including tardive dyskinesia (TD), Alzheimer’s disease (AD) and senile dementia (Ferris et al., 1977; Penovich et al., 1978; de Montigny et al., 1979; Fisman et al., 1981; George et al., 1981). First, application of DMEA to human healthy volunteers dates back to the 1960s when DMEA was reported to exert stimulating effects comparable to amphetamine (Murphree et al., 1960; Pfeiffer et al., 1963). Murphree et al. (1960) described improved concentration, increased muscle tone and changed sleeping habits in healthy males (21–26 years) with an intake of 10–20 mg DMEA (or Deanol) daily for 2–3 weeks compared to a placebo group. In later studies, DMEA was hypothesized as an acetylcholine (ACh) precursor and therefore tested in diseases that are considered to be linked to the cholinergic system. However, results of several studies were inconclusive and a systematic review could not confirm the positive effects of DMEA or other cholinergic compounds in patients with TD (Tammenmaa et al., 2004). In addition, *in vivo* experiments showed that DMEA is not methylated to choline and does not alter brain ACh levels (Millington et al., 1978; Jope and Jenden, 1979).

Interestingly, in both acute and chronic seizure models in rats, a conjugate of DMEA and valproate (DEVA) was shown to be more potent than valproate alone, potentially by facilitation of valproate transport *via* the blood brain barrier (Shekh-Ahmad et al., 2012). In this study, however, the effects of DMEA alone were not tested. To our knowledge, effects of DMEA on pathological neuronal network activity have never been investigated before.

In principle, resected human tissue of temporal lobe epilepsy (TLE) patients carries the potential to bridge the translational gap between preclinical and clinical drug development. Animal models have been instrumental in the discovery and preclinical development of novel AEDs (Löscher, 2011). However, animal models cannot represent all aspects of complex neurological disorders and sometimes produce misleading results as exemplified by the neuropeptide galanin. Galanin showed robust antiepileptic effects in a mouse model of epilepsy, however, the effect could not be reproduced in resected human tissue (Ledri et al., 2015).

Here, we decided to investigate the effects of DMEA on epileptiform activity directly in *ex vivo* human tissue resected from epilepsy patients.

## Materials and Methods

### Primary Rat Hippocampal Neuronal Cell Culture

Hippocampal cultures from E18 Wistar rat embryos were prepared as previously described (Winkelmann et al., 2014) according to the approval by the Animal Care Committee of the Technical University Braunschweig (Zentrale Einrichtung für Tierhaltung der TU Braunschweig, §4 10.15.R TSB TU BS) and maintained in Neurobasal medium supplemented with B27 and 1% FCS (Brewer et al., 1993). Hippocampal neurons were subjected to whole cell patch clamp analysis at DIV13–16 to ensure that the cultures were mature enough to display synaptic activity.

### Electrophysiological Recordings of Cultured Hippocampal Neurons

An EPC-7 amplifier (List-Medical, Darmstadt, Germany), ITC-18 interface and Patchmaster software (both HEKA, Lamprecht, Germany) were used for patch clamp recordings and data acquisition. Patch pipettes made from borosilicate glass (Science Products, Hofheim, Germany), had resistances of 4–7 MΩ when filled with the intracellular solution containing (in mM): KCl (130), NaCl (5), CaCl_2_ (0.5), MgCl_2_ (1), EGTA (5) and HEPES (30), pH 7.2 (KOH). Neurons were continuously perfused with carbogenated artificial cerebrospinal fluid (aCSF) containing (in mM): NaCl (125), KCl (2), MgCl_2_ (1), CaCl_2_ (2), NaHCO_3_ (25), NaH_2_PO_4_ (1.25) and glucose (10), pH 7.4. Extracellular solution was supplied by gravity using a perfusion pencil (Automate Scientific Inc., Berkeley, CA, USA) for fast solution exchange. The extracellular solution was supplemented with 0.5, 2, 5 or 10 mM DMEA. Series resistances (Rs) were monitored by 5 mV voltage pulses (50 ms) applied every 5 s and varied between 10 MΩ and 30 MΩ. Data were acquired with a sampling rate of 20 kHz and filtered at 2.8 kHz. All experiments were carried out at room temperature (~24°C).

The effect of DMEA on synaptic activity and excitability was tested in current-clamp mode with stepwise increase of DMEA concentration. DMEA was applied for 60 s, following 3–5 min wash-out with aCSF before applying the next drug concentration. No synaptic or intrinsic blockers were added. A steady current injected into the cell was used to hold the membrane potential around −50 mV never exceeded 150 pA. Action potential (AP) threshold was investigated by short current injections using 10 pA steps with or without the application of DMEA. AP threshold was determined as the mean between the minimum membrane potential necessary for AP generation and membrane potential of the previous step current.

### Human Tissue Transport and Preparation

Human hippocampal tissue from epilepsy surgery was collected from 12 TLE patients (10 males, two females), who all gave written consent prior to the procedure. Experiments were approved by the Ethics Committee of Charité-Universitätsmedizin Berlin on the 1st of November 2014 (EA2/111/14) and performed in agreement with the Declaration of Helsinki.

Hippocampal tissue was transferred to carbonated (95% O_2_, 5% CO_2_), ice-cold transport solution immediately after resection. Tissue was transported to the lab in <60 min and cut to 400 μm slices using a vibratome (Leica VT1200S, Wetzlar, Germany). N-methyl d-glucamine (NMDG)-aCSF was used for brain tissue transport and slice preparation from patients 1–7, while choline-aCSF was used to handle tissue of patient 8–12 in order to optimize transport. NMDG-aCSF contained (in mM): NMDG (93), KCl (2.5), NaH_2_PO_4_ (1.2), NaHCO_3_ (30), MgSO_4_ (10), CaCl_2_ (0.5), HEPES (20), glucose (25), Na-L-ascorbate (5), thiourea (2), Na-pyruvate (3; Ting et al., 2014). Choline-aCSF contained (in mM): Choline chloride (110), KCl (2.5), NaH_2_PO_4_ (1.25), NaHCO_3_ (26), MgCl_2_ (7), CaCl_2_ (0.5), glucose (10), Na-L-ascorbate (11.6), Na-pyruvate (3.1; Testa-Silva et al., 2010).

As part of the standard clinical routine, every third slice collected during the slicing procedure was fixed in 4% paraformaldehyde (PFA, pH 7.4, phosphate-buffered saline) and analyzed for pathological alterations. For electrophysiological recordings, slices were stored in an interface chamber for a recovery period of at least 5 h and continuously perfused with carbogenated aCSF containing (in mM): NaCl (129), NaH_2_PO_4_ (1.25), CaCl_2_ (1.6), KCl (3.0), MgSO_4_ (1.8), NaHCO_3_ (21), Glucose (10; 1.6 ml/min, 35°C, pH 7.4). All slices were studied between 6 h and 20 h after resection.

### Electrophysiological Recordings of Human Tissue

For electrophysiological recordings, slices were transferred to a custom modified version of the membrane chamber (Hill and Greenfield, 2011) and perfused with aCSF (10 ml/min, 32°C). The membrane chamber is a submerged-type recording chamber with a high flow rate but stable slice position, guaranteeing optimal oxygen supply and fast drug applications. Field potential recordings were performed with borosilicate pipettes (1.5 mm outer diameter, Science Products, Hofheim, Germany) pulled with a vertical puller (1–2 MΩ, PC-10, Narishige, Tokyo, Japan) and filled with NaCl (154 mM). Signals were recorded from CA1 pyramidal cell layer, sampled at 10 kHz, low-pass filtered at 2 kHz by a Digidata 1550 interface and processed by PClamp10 software (Molecular Devices, Sunnyvale, CA, USA).

Induction of epileptiform activity in rodent brain slices can be achieved by a stand-alone manipulation such as inhibition of potassium channels by 4-aminopyridine (4-AP; Perreault and Avoli, 1991; Avoli et al., 2002; Heuzeroth et al., 2019). On the contrary, induction of epileptiform activity in human brain tissue *ex vivo* is less straightforward and subject to controversy. In resected human brain tissue only application of 4-AP in combination with electrical stimulation or elevated extracellular potassium was able to show stable induction of epileptiform activity, not application of 10 or 12 mM potassium or 4-AP alone (Gabriel et al., 2004; Antonio et al., 2016). Here, we used bath application of high potassium (8 mM) and 4-AP (100 μM, Sigma, Munich, Germany) for the induction of epileptiform activity in human brain tissue. Increase of osmolarity by 8 mM KCl was balanced by lowering NaCl concentration from 129 mM to 124 mM.

Baseline epileptiform activity was recorded for ≥20 min. DMEA (10 mM, Sigma, Munich, Germany) or lacosamide (LAC, 100 μM, Biozol, Eching, Germany) were then applied for ≥20 min, followed by ≥20 min of wash-out. In initial experiments we tested 5 mM and 10 mM of DMEA in the same slice, starting with 5 mM DMEA (20 min), followed by 10 mM DMEA and wash-out as described above.

Addition of DMEA increases osmolarity of aCSF possibly affecting neuronal activity. Therefore, we performed control experiments with increasing the osmolarity of aCSF up to ~310 mOsm by addition of 10 mM sucrose (patients 10, 11, and 12). This approach has been used to test hyperosmolar solutions before (Rosenmund and Stevens, 1996). Here, the recording sequence stated above was modified and consisted of the following steps: (1) baseline with stable epileptiform activity (≥10 min); (2) 10 mM sucrose (≥20 min); and (3) wash-out sucrose (≥10 min), 10 mM DMEA (≥20 min), wash-out DMEA (≥20 min).

### Data Analysis and Statistics

Electrophysiological data recorded in single cells from primary neuronal cell cultures was quantified and measured using an IGOR Pro (Version 6.3.7.2, Wavemetrics Inc., Oregon, USA) procedure written by Dr. Marcus Semtner (MDC Berlin) and the extension PatchersPowerTools (written by Dr. Francisco Mendez and Frank Würriehausen).

Recordings in human hippocampal tissue were band pass filtered (1–1,000 Hz) and the 300 s long episodes (last 5 min of each application phase) were analyzed with Clampfit 10.7 (Molecular Devices, Sunnyvale, CA, USA) threshold analysis. All events visually identified as burst activity (defined by biphasic, positive and negative deflection and a duration ≥100 ms) were manually indicated for further analysis of event frequency (inter-event-interval, IEI), amplitude and total number of events during the analyzed time frame. Interictal spikes (defined by exclusive negative deflections and a duration <10 ms) were not analyzed. According to literature, interictal spikes, although pathologically relevant, are not significantly affected by AEDs (Spencer et al., 2008; D’Antuono et al., 2010).

All data were analyzed with GraphPad Prism 5 (GraphPad Software Inc., San Diego, CA, USA). Prior to analysis, data were subjected to D’Agostino and Pearson omnibus normality test and analyzed accordingly. In cases where sample size of tested groups was too small for evaluation of data distribution, data were analyzed using non-parametric tests.

Statistical analysis of normal distributed data was performed using repeated measurement analysis of variance (ANOVA) and *post hoc* Tukey’s comparison of all groups. Non-normal distributed data or data with small sample size was analyzed either using Friedman test and *post hoc* Dunnett’s multiple comparison of individual groups or Wilcoxon signed-rank test. For all analysis, a *p*-value < 0.05 was considered statistically significant.

Data analyzed by parametric tests were presented as scatter plots with mean ± standard deviation (SD) while data analyzed by non-parametric tests were presented as scatterplots with median and interquartile range. Due to IEI variance during drug application (DMEA or LAC), frequency of events for all patients was summarized as number of events. IEI for individual recordings is presented as boxplots with median and Tukey whiskers (1.5× the interquartile distance) to show data distribution and inter-individual differences.

Human datasets analyzed in this study can be found in figshare repository (https://doi.org/10.6084/m9.figshare.8148572).

## Results

**Figure 1:**
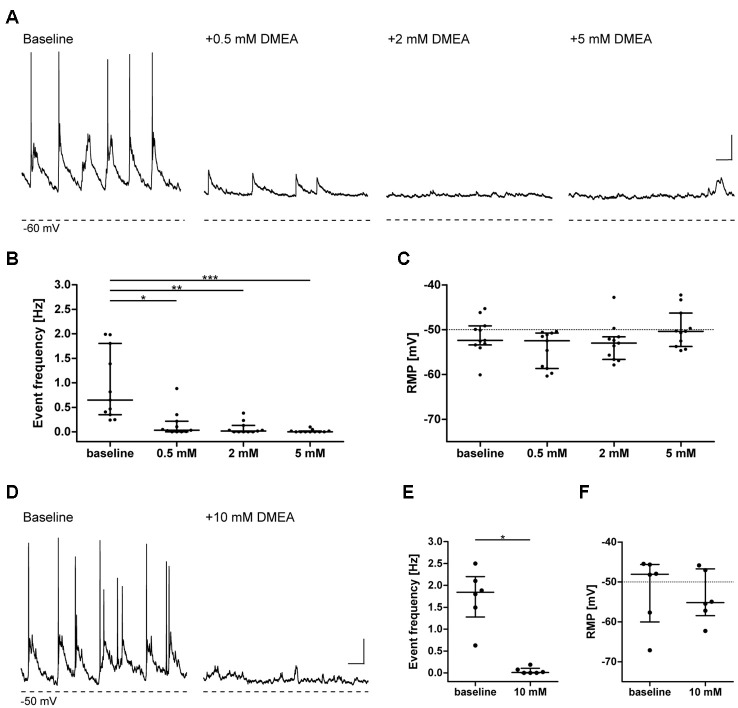
Dimethylethanolamine (DMEA) decreases spontaneous neuronal activity in neuronal cultures in a concentration-dependent manner. Activity of cultured hippocampal neurons (DIV 13–16) was investigated in current-clamp mode in the absence or presence of DMEA. During baseline conditions, all neurons displayed excitatory synaptic events with associated action potentials (APs) at a frequency of ~1 Hz. Both AP, as well as large amplitude synaptic event frequency, decreased with increasing DMEA concentration to reach a full block with 5 mM DMEA. **(A,D)** Exemplary recordings in neurons with application of 0.0, 0.5, 2.0 and 5.0 mM DMEA **(A)** or 10 mM DMEA **(D)**; note that due to experimental setup we could not perform repeated measurement in one cell including all concentrations and present the results here separately; Dashed lines below recordings indicate resting membrane potential (RMP, in mV) of cells. **(B,E)** DMEA significantly decreases event frequency in a concentration-dependent manner. **(C,F)** Application of DMEA does not affect RMP. All data are shown as scatter plots with median and interquartile range. Asterisks mark significant differences as assessed by Friedman test and *post hoc* by Dunnett’s multiple comparison of groups **(B,C)** or by Wilcoxon signed-rank test (**E,F**; *p < 0.05, **p < 0.01, ***p < 0.001). Scale bar: 10 mV, 0.5 s.

To investigate whether DMEA affects neuronal activity and to obtain information about potential molecular targets, we performed electrophysiological recordings in primary neuronal cultures. DMEA dose-dependently reduced spontaneous synaptic events and associated APs with an estimated EC50 of ~0.5–1.0 mM. A full block of activity was observed in all recorded cells when using 5 mM or 10 mM DMEA (Figures 1A,B,D,E). Neither resting membrane potential (RMP) nor AP threshold changed upon DMEA application (Figures 1C,F, Supplementary Figures S1A,B), suggesting that intrinsic properties do not represent the main target of DMEA action.

We also investigated the effects of DMEA on epileptiform activity in hippocampal tissue resected from 12 patients undergoing epilepsy surgery. Histopathology of resected tissue revealed distinct pathologies: malformations of cortical development (MCD) were diagnosed in two patients [mild MCD changes in one and clear focal cortical dysplasia (FCD) in the other patient], unspecific astrogliosis in three patients and clear pathological changes indicating hippocampal sclerosis (HS) in seven patients (Supplementary Table S1). The changes in pyramidal layers of CA1, CA3 and CA4 included <10% neuronal cell loss in three patients (Wyler grade 1), up to 50% cell loss in three patients (Wyler grade 2) and more than 50% cell loss in one patient (Wyler grade 3).

**Figure 2:**
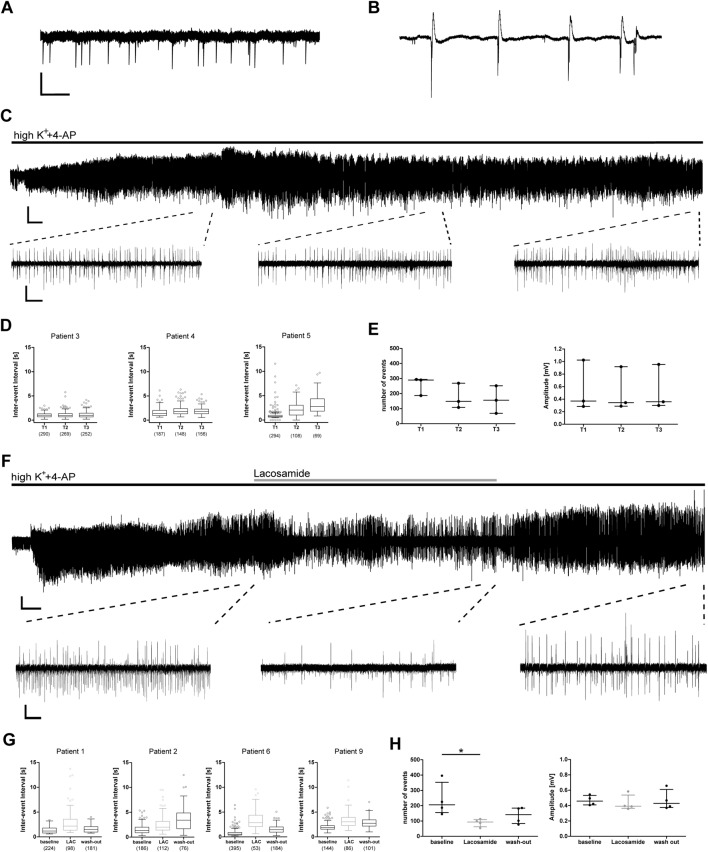
Epileptic burst activity in human slices is stable over long time periods and decreases during application of lacosamide. Application of 8 mM K^+^ and 100 μM 4-aminopyridine (4-AP) induces two patterns of network activity recorded by field potential electrodes in the CA1 area of human hippocampal slices: interictal spikes **(A)** and burst activity **(B)**. **(C–E)** Burst activity is induced a few minutes after application and stable for at least 60 min. **(F–H)** Induced burst activity decreases during application of lacosamide (LAC). **(C,F)** Exemplary recording with excerpts of regions used for analysis. **(D,G)** Inter-event intervals (IEI) of individual recordings shown as box plots with mean and 1.5× interquartile distance. Each dot represents data point outside the 1.5× interquartile distance. Total number of IEI during analyzed time frame are indicated in brackets. **(E,H)** Summarized results for all patients [number of events and amplitude as mean ± standard deviation (SD)]; each dot indicates one patient. Asterisks mark significant differences as assessed by Friedman test and *post hoc* with Dunnett’s multiple comparison of groups (*p* < 0.05). T1, T2, T3 in **(D,E)** are periods analyzed for each patient, comparable to analyzed times in **(G,H)** and Figure 3. Scale bars: 0.2 mV, 500 ms **(A,B)**, 5 min (full recording, **C**), 2 min (full recording, **F**), 5 s (excerpts, **C,F**).

From these 12 resected samples, a total of 30 hippocampal slices was used for electrophysiological recordings. After slice recovery, we tested our experimental approach by assessing whether: (1) epileptiform activity in human slices can be induced by elevated potassium and 4-AP; (2) whether this activity is stable for prolonged periods of time; and (3) sensitive to conventional AEDs. In 27 slices from 12 patients, both interictal spikes and ictal burst activity (Figures 2A,B) could be induced within a few minutes (Figures 2C,F). In three slices from three patients, burst events did not occur within 20 min, therefore these slices were excluded from the analysis.

In control recordings without drug application, the pathologically relevant burst activity was stable for at least 60 min (tested in three slices from three patients), suggesting good viability of human slices in our experimental setting. In more detail, both frequency and amplitude of burst activity did not change in most cases during the recording as indicated by stable IEI, amplitude and number of burst events (Figures 2D,E).

To test whether burst activity was sensitive to conventional AEDs, we investigated the effect of the sodium channel blocker LAC. During application of LAC, IEI increased in all recorded slices, which was reversible in three out of four patients (Figure 2G). Number of events of all recordings decreased during LAC application when compared to baseline activity, while amplitude of burst activity did not change (Figure 2H). In conclusion, we were able to induce stable epileptiform activity that can be inhibited by clinically approved AEDs.

**Figure 3:**
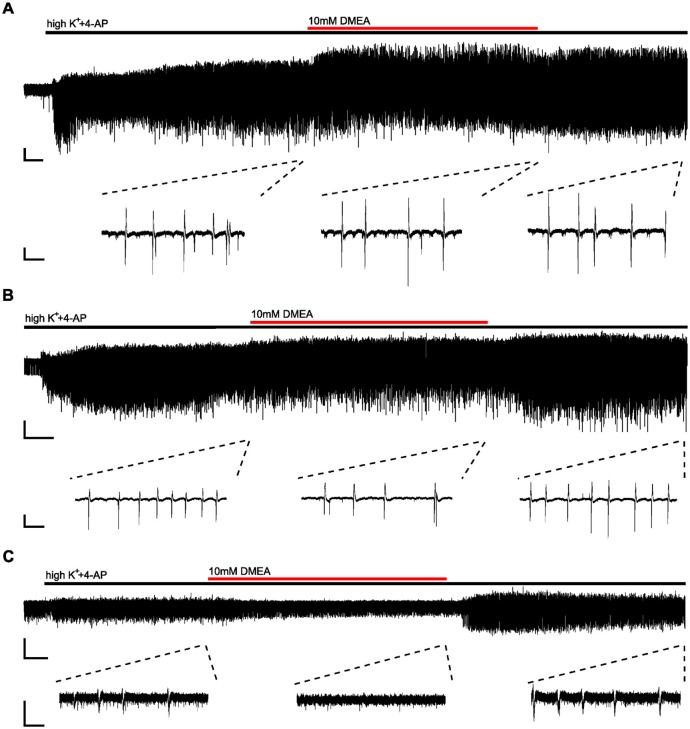
Effects of DMEA on epileptiform activity in human brain tissue *ex vivo*. Example traces displaying variability of DMEA effects on burst activity; full recordings (top) and excerpts (bottom) from the end of each period (baseline, DMEA and wash-out) demonstrate different effects of DMEA including a lack of response (**A**, patient 8), a partial block (**B**, patient 7) and a full block (**C**, patient 11). Scale bars: 0.2 mV, 3 min (full recordings), 1 s (excerpts).

**Figure 4:**
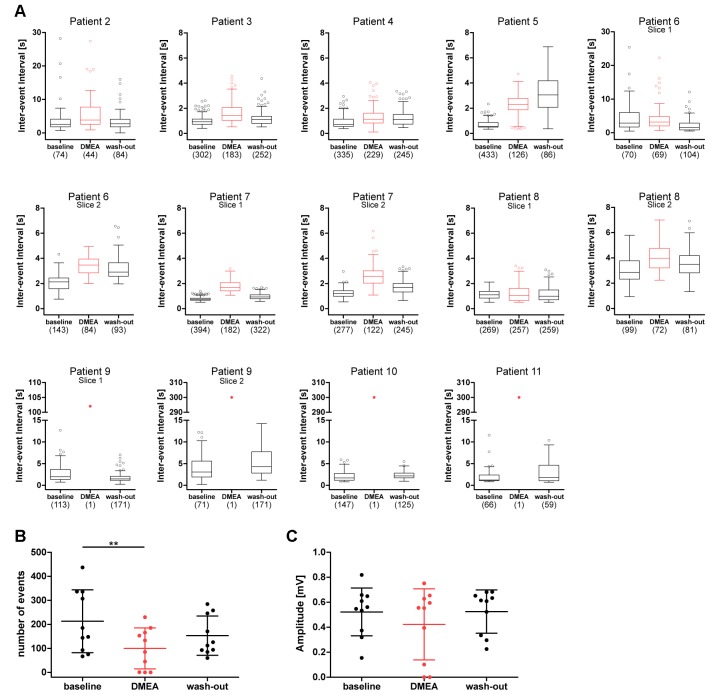
DMEA reduces epileptiform activity in human hippocampal slices. Summary of DMEA effects on burst activity recorded in CA1 pyramidal cell layer of resected human hippocampus. **(A)** Box plots of IEIs with mean and 1.5× interquartile distance before, during and after DMEA application for each patient are shown for all recorded slices (*n* = 10 patients). Dots represent data points outside the 1.5× interquartile distance. Total number of IEI during analyzed time frame are indicated in brackets. **(B)** Summary of DMEA effects on number and **(C)** amplitude of burst events for all patients; each dot indicates one patient. Data is presented as scatter plots with mean ± SD, asterisks mark significant differences as assessed by repeated measurement analysis of variance (ANOVA) and *post hoc* with Tukey’s comparison (**p < 0.01, *n* = 10).

Next, we investigated the effects of DMEA in 14 hippocampal slices from 10 TLE patients. In initial experiments, we tested the effects of 5 mM and 10 mM DMEA within one experiment (Supplementary Figure S2). As the inhibitory effect was more robust when using 10 mM DMEA (Supplementary Figure S2B, *p* < 0.05, *n* = 6; tested in patients 2–7), we decided to perform subsequent experiments with this effective concentration. In contrast to LAC, DMEA effects varied considerably between samples ranging from no effect (patient 8, Figure 3A) or only a moderate effect observed in slices of patient 7 (Figure 3B) to a full block of burst activity (Figure 3C) in four slices from patients 9, 10 and 11. Irrespective of the effect variability, DMEA displayed antiepileptic effects in 10 out of 11 patients (Figure 4A), as indicated by the increase of IEI and a significant decrease of number of events during application of DMEA (Figure 4B, *p* < 0.01, *n* = 10). In eight patients, these effects were clearly reversible upon wash-out of DMEA. Amplitude of epileptiform bursts was not affected (Figure 4C).

Addition of DMEA at 10 mM increases osmolarity of the extracellular environment (here, aCSF) which could possibly decrease epileptiform activity simply by osmotic effects (Traynelis and Dingledine, 1989; Dudek et al., 1990). To test whether DMEA effects are mediated by mechanisms beyond pure change in osmolarity, we investigated how sucrose and DMEA affect epileptiform activity in the same slice when applied sequentially. In six slices of three patients increase of osmolarity by sucrose (310 ± 5 mOsm) to match osmolarity of solutions containing 10 mM DMEA resulted in a weak increase of IEI and a weak decrease of number of events (Supplementary Figure S3). This decrease matched the observed decrease without intervention over time as shown in Figure 2E and was likely due to a slow decay in activity in the course of *in vitro* experiments. The mild effect of sucrose application stood in contrast to a full block of activity during subsequent application of DMEA in patients 10 and 11 and a strong decrease of activity in patient 12 (Supplementary Figures S3A,B).

In summary, in human brain slices DMEA exerted antiepileptic effects presented as an overall decrease in frequency of burst events, which were not due to osmotic changes. A summary of all results is presented in Supplementary Table S2.

## Discussion

In the present study, we investigated the effect of DMEA on spontaneous activity in neuronal cultures and on epileptiform activity in human hippocampal tissue *ex vivo*. We were able to show a decrease of spontaneous activity in rodent neuronal cultures. In the majority of investigated human slices, DMEA displayed strong antiepileptic effects including full block of burst activity in slices of three patients. The effect of DMEA in human tissue varied considerably, implying interindividual differences in expression patterns of molecular DMEA targets.

The mechanism of DMEA action is not known. Our preliminary experiments in neuronal cultures showing dose-dependent inhibition of spontaneous neuronal activity without altering RMP or AP threshold suggest that DMEA likely affects synaptic but not intrinsic excitability. In previous clinical studies, DMEA was tested as a potential ACh precursor but did not improve disease symptoms in TD or AD. DMEA increased choline concentrations in plasma and brain though this did not affect brain ACh concentrations, questioning a beneficial effect of DMEA in diseases involving the cholinergic system (Millington et al., 1978; Jope and Jenden, 1979). Nevertheless, due to structural similarities of DMEA, ACh and choline, muscarinic ACh receptors (mAChRs) present possible targets of DMEA action. ACh and choline are both ligands of M2 and M3 mAChRs (Shi et al., 2004; Moreno-Galindo et al., 2016), which upon activation increase the open probability of G-protein coupled inwardly rectifying potassium (GIRK) channels, specifically GIRK1 and GIRK4 (Nemec et al., 1999). GIRK1 and GIRK4 are expressed in the hippocampus (Miyashita and Kubo, 1997; Murer et al., 1997; Cea-del Rio et al., 2010) and pharmacological activation of GIRK results in antiepileptic effects *in vitro* and *in vivo* (Kaufmann et al., 2013). Thus, M2 or M3 mAChRs and GIRK channels present a possible mechanism for DMEA action.

Another possible candidate are glycine receptors (GlyR). In a separate project, our group analyzed the effects of DMEA on GlyR, which has been described as subject to increased RNA editing in TLE (Krestel et al., 2013; Winkelmann et al., 2014; Meier et al., 2016; Srivastava et al., 2017). Presynaptic RNA-edited GlyR facilitates neurotransmitter release resulting in increased neuronal gain and, depending on the neuronal subtype, network hyper- or hypoexcitability (Winkelmann et al., 2014; Caliskan et al., 2016). In an initial screen for antagonists against RNA-edited GlyR (data not shown), DMEA was considered as a candidate giving an additional incentive to test this substance in human brain slices. However, confirmatory experiments were not able to reproduce DMEA specificity against RNA-edited GlyR. Overall, the molecular targets of DMEA and its mechanism of action in brain tissue remain indeterminate.

The main goal of our study was to demonstrate a possibly clinically relevant effect of DMEA. DMEA has been tested in patients and healthy volunteers since 1960s (Murphree et al., 1960; Pfeiffer et al., 1963). In healthy males, Murphree et al. (1960) reported no change in heart rate, body weight, muscle power, hand steadiness or vital capacity with an intake of 10–20 mg DMEA (or Deanol) daily for 2–3 weeks. In two double-blinded, placebo-controlled studies of TD, DMEA led to side effects such as lethargy, drowsiness and a mild but significant increase in the schizophrenia score (de Montigny et al., 1979; George et al., 1981), although a systematic review was not able to confirm an increased risk for psychosis (Tammenmaa et al., 2004). Fisman et al. (1981) detected severe neurological and cardiovascular effects (apathy, motor retardation, increased confusion associated with rise in systolic and diastolic blood pressure) in two patients with AD when treated with 1,800 mg DMEA daily. In these studies, as compared to Murphree et al. (1960), DMEA was administered in a 100-fold higher daily dosage (10–20 mg vs. 1,000–2,000 mg). A dose of 2,000 mg DMEA corresponds to a molar amount of 22 mM and a final *in vivo* concentration of 0.44 mM assuming a distribution across all compartments and a body water content of approximately 50 l for an adult male. This concentration is lower by a factor of 20 when compared to 10 mM tested to be effective in human brain tissue *ex vivo* in our study, and this difference is rather underestimated when taking into account oral bioavailability. Of note, in neuronal cultures, the effective concentration to inhibit neuronal activity was lower by a factor of 10, implying that differences in microenvironment, neuronal connectivity and drug diffusion in different preparations and species might influence the effective concentration.

Addition of DMEA to our experimental solution increased the osmolarity by ~10 mOsm. In previous studies, application of hyperosmolar solutions decreased neuronal excitability and epileptiform activity, but in most cases, an increase of 30 mOsm and more was necessary to induce these effects (Traynelis and Dingledine, 1989; Dudek et al., 1990; Rosen and Andrew, 1990). According to Rosen and Andrew (1990), an increase of extracellular osmolarity by 10 mOsm might result in a 10% decrease of EPSC amplitude in CA1 pyramidal neurons, but the effects of such EPSC alteration on network activity have not been investigated. Here, increase of aCSF osmolarity by application of 10 mM sucrose did not alter epileptiform activity, indicating that the antiepileptic effects of DMEA are not primarily mediated by changes in osmolarity.

Although our study clearly demonstrated an antiepileptic effect of DMEA, the large concentration difference between our work and previous clinical studies and the variability of underlying pathologies represent substantial limitations. The results suggest heterogeneous expression of DMEA targets, however, also differences in neuronal survival and the degree of HS could possibly contribute. An additional limitation is given by different transport solutions used in our study, namely either NMDG or choline aCSF (Testa-Silva et al., 2010; Eyal et al., 2018; Ting et al., 2018). Although we were unable to detect differences in tissue quality as seen by stable induction and properties of epileptiform activity, we cannot exclude differences in neuronal survival between these solutions.

The predictive value of brain activity for drug development in an *ex vivo* setting is still unclear. In our approach using a modified submerged chamber, we mostly observed burst activity (spike and burst activity) described before as interictal-like (Remy et al., 2003; Gabriel et al., 2004; Sandow et al., 2015; Reyes-Garcia et al., 2018) and standing in contrast to ictal discharges lasting >10 s. Brückner and Heinemann (2000) suggested that AEDs applied in brain slices (of non-epileptic animals) rather inhibit ictal but not interictal activity. Other animal studies *in vitro*, however, demonstrated a robust decrease in interictal activity in the hippocampus upon AED application (Fueta and Avoli, 1992; Holtkamp et al., 2017; Taing et al., 2017), which was comparable to our results with LAC and DMEA. We show that burst activity was sensitive to the approved antiepileptic LAC, indicating that our experimental setting is capable of validating and possibly predicting antiepileptic efficacy for drug development.

Taken together, our work identified the antiepileptic effect of DMEA in human hippocampal tissue from TLE patients. Interindividual effect variability, differences in the effective concentration range and previously reported side effects call for future, more detailed investigation of cell-type specific effects and molecular targets of DMEA.

## Data Availability

All datasets generated for this study are included in the manuscript and/or the Supplementary Files. Human datasets analyzed in this study can be found in the figshare repository (https://doi.org/10.6084/m9.figshare.8148572).

## Ethics Statement

The studies involving human participants were reviewed and approved by Charité-Universitätsmedizin Berlin (EA2/111/14). The patients/participants provided their written informed consent to participate in this study. The animal study was reviewed and approved by Animal Care Committee of the Technical University Braunschweig (Zentrale Einrichtung für Tierhaltung der TU Braunschweig, §4 10.15.R TSB TU BS).

## Author Contributions

JM and PF designed the study. MH selected patients for operation. US operated patients. HR performed histopathological analysis. LK performed and analyzed recordings in human tissue. FH performed neuronal experiments. LK and FH analyzed neuronal experiments. LK and PF wrote the manuscript, which all authors edited and finalized.

## Conflict of Interest Statement

The authors declare that the research was conducted in the absence of any commercial or financial relationships that could be construed as a potential conflict of interest.

## Supplementary Material

FIGURE S1 DMEA does not change action potential threshold. Application of 10 mM DMEA does not change threshold for AP generation. **(A)** Exemplary recordings of patch-clamp recordings in current-clamp mode with step current without AP (first for each) and with AP generation (second); dashed lines below recordings show −60 mV value to indicate RMP of cells. **(B)** Quantified effect of DMEA on AP threshold in neurons. Data are shown as median with interquartile range and was subjected to Wilcoxon signed-rank test; *n* = 6.

FIGURE S2 Dose-dependent effects of DMEA on epileptiform activity in human hippocampal slices. **(A)** Box plots of inter-event intervals (IEI) during baseline, 5 mM DMEA, 10 mM DMEA and wash-out for each patient given for all recorded slices (*n* = 6 patients). In all experiments, both concentrations were applied in the same brain slice. Box plots are shown with mean and Tukey whiskers (1.5× interquartile distance). Each dot represents a data point outside the 1.5× interquartile distance. Total number of IEI during analyzed time frame are indicated in brackets. **(B)** Summary of DMEA effects on the number and amplitude of burst events for all tested patients; each dot indicates one patient. Data are presented as scatter plots with median ± interquartile range. Asterisks mark significant differences as assessed by Friedman test and *post hoc* with Dunnett’s multiple comparison of groups (**p* < 0.05).

FIGURE S3 Increase of osmolarity by 10 mOsm does not affect epileptiform activity. For control purposes, sucrose was used to increase osmolarity and applied in the same slice before DMEA application. **(A)** Full time course and detailed excerpts of exemplary recording used for the analysis of potential hyperosmotic effects of DMEA on epileptiform activity. Note that sucrose application leading to the same osmotic change as DMEA does not significantly alter epileptiform activity, while DMEA application in the same slice clearly exerts an antiepileptic effect. **(B)** Inter-event intervals of individual recordings shown as box plots with a total number of IEI during analyzed time frames indicated in brackets. **(C)** Summarized number of events and amplitudes for all patients presented as scatterplots with median and interquartile range; each dot indicates one patient. Scale bars: 0.2 mV, 5 min (full recordings), 5 s (excerpts).

TABLE S1 Clinical data of patients, who provided tissue for electrophysiological assessment of the effects of DMEA on epileptic activity.

TABLE S2 Summarized results of burst activity in human tissue during application of DMEA, LAC, no intervention or sucrose as control for osmolarity effects.
