# Supplementary figures and images for: Dimethylethanolamine Decreases Epileptiform Activity in Acute Human Hippocampal Slices in vitro
Source: Front Mol Neurosci. 2019 Sep 6;12:209. doi: 10.3389/fnmol.2019.00209 (PMC6743366; doi:10.3389/fnmol.2019.00209)

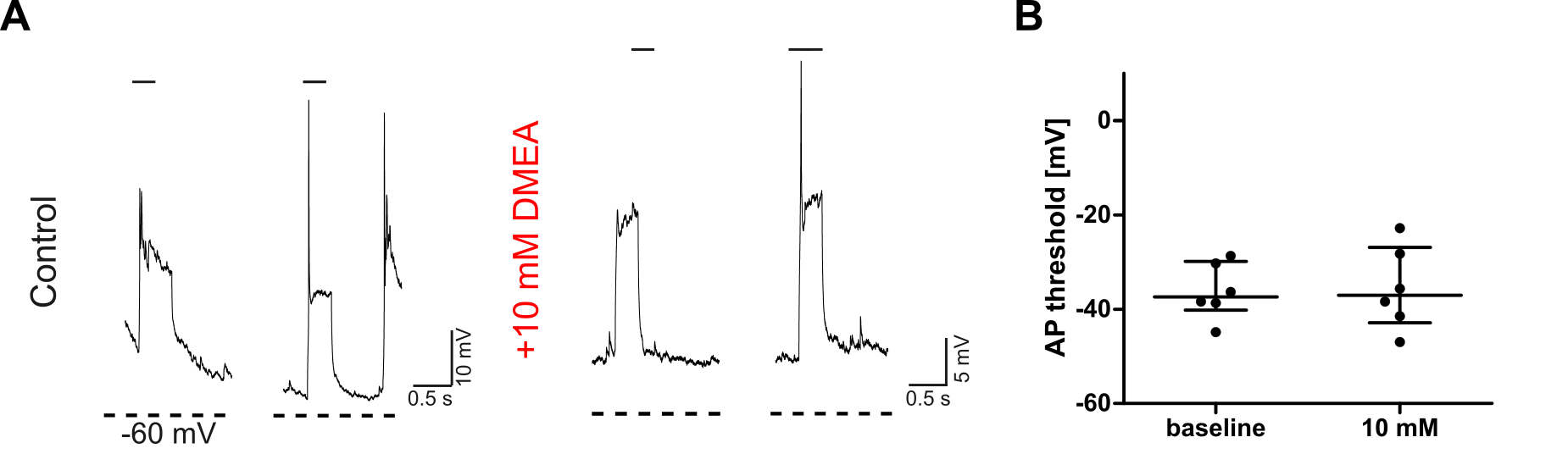

Supplement: FIGURE S1 — DMEA does not change action potential threshold. Application of 10 mM DMEA does not change threshold for AP generation. (A) Exemplary recordings of patch-clamp recordings in current-clamp mode with step current without AP (first for each) and with AP generation (second); dashed lines below recordings show −60 mV value to indicate RMP of cells. (B) Quantified effect of DMEA on AP threshold in neurons. Data are shown as median with interquartile range and was subjected to Wilcoxon signed-rank test; n = 6. [file Image_1.TIF]

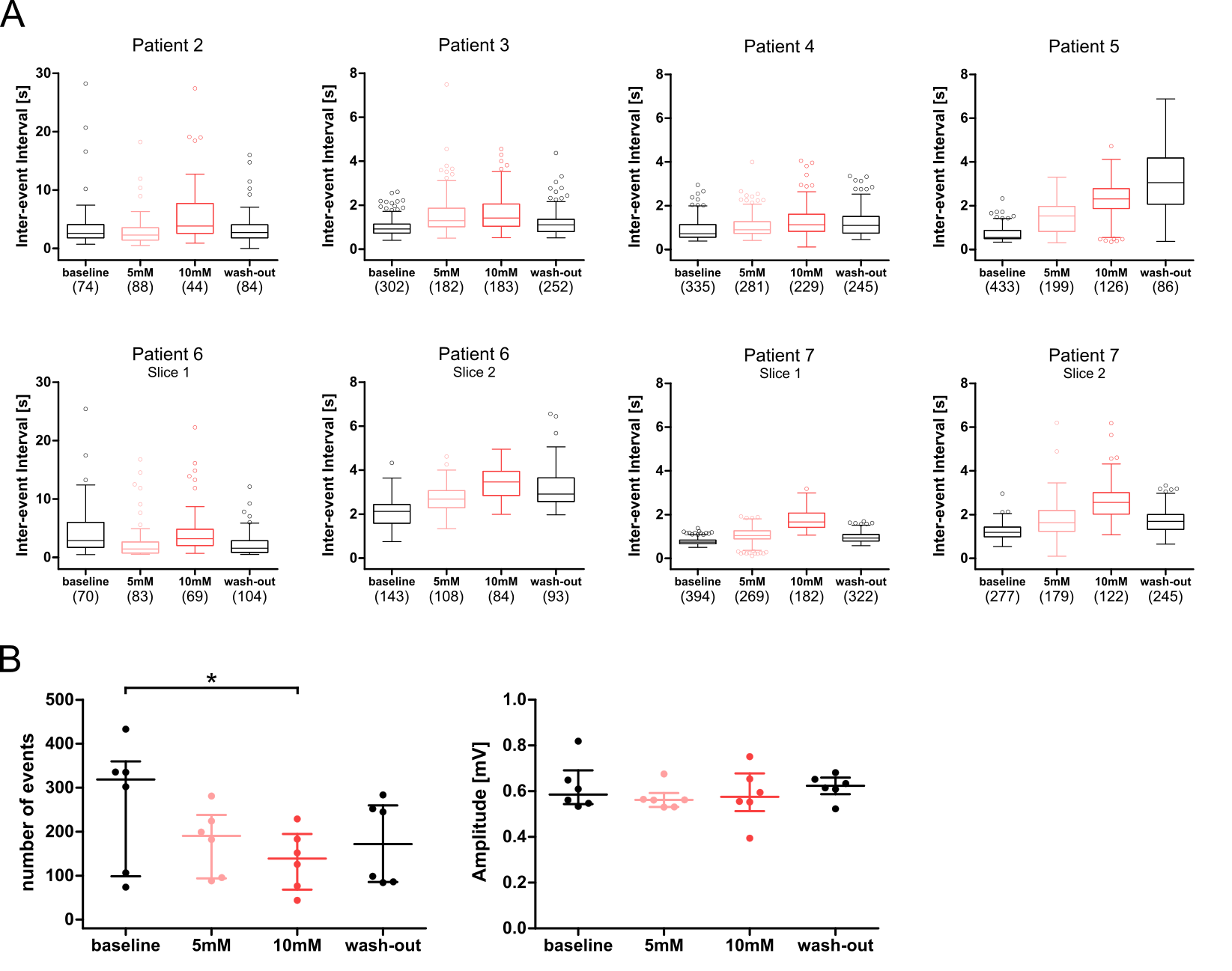

Supplement: FIGURE S2 — Dose-dependent effects of DMEA on epileptiform activity in human hippocampal slices. (A) Box plots of inter-event intervals (IEI) during baseline, 5 mM DMEA, 10 mM DMEA and wash-out for each patient given for all recorded slices (n = 6 patients). In all experiments, both concentrations were applied in the same brain slice. Box plots are shown with mean and Tukey whiskers (1.5× interquartile distance). Each dot represents a data point outside the 1.5× interquartile distance. Total number of IEI during analyzed time frame are indicated in brackets. (B) Summary of DMEA effects on the number and amplitude of burst events for all tested patients; each dot indicates one patient. Data are presented as scatter plots with median ± interquartile range. Asterisks mark significant differences as assessed by Friedman test and post hoc with Dunnett’s multiple comparison of groups (*p < 0.05). [file Image_2.TIF]

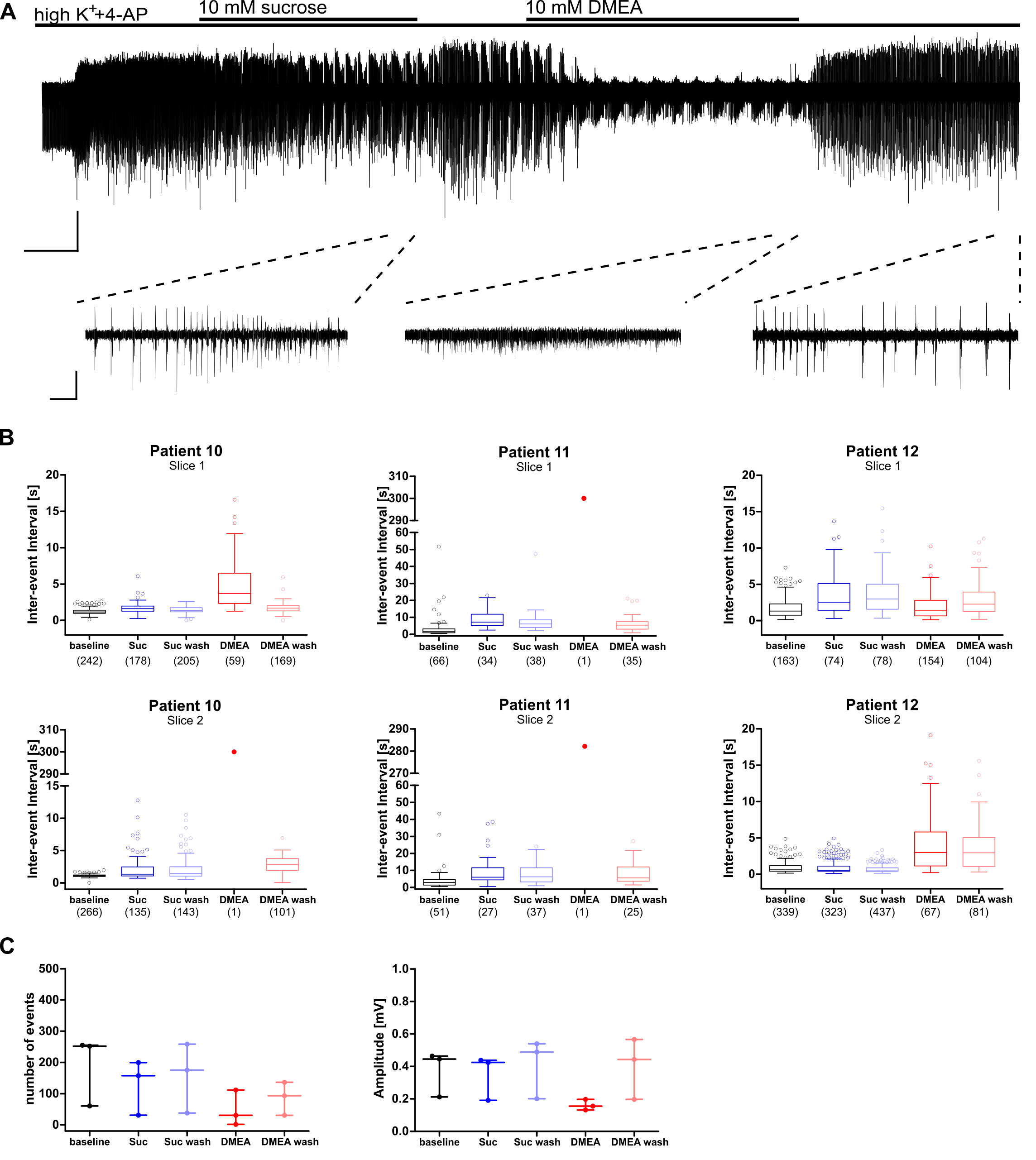

Supplement: FIGURE S3 — Increase of osmolarity by 10 mOsm does not affect epileptiform activity. For control purposes, sucrose was used to increase osmolarity and applied in the same slice before DMEA application. (A) Full time course and detailed excerpts of exemplary recording used for the analysis of potential hyperosmotic effects of DMEA on epileptiform activity. Note that sucrose application leading to the same osmotic change as DMEA does not significantly alter epileptiform activity, while DMEA application in the same slice clearly exerts an antiepileptic effect. (B) Inter-event intervals of individual recordings shown as box plots with a total number of IEI during analyzed time frames indicated in brackets. (C) Summarized number of events and amplitudes for all patients presented as scatterplots with median and interquartile range; each dot indicates one patient. Scale bars: 0.2 mV, 5 min (full recordings), 5 s (excerpts). [file Image_3.TIF]
