# Supplementary material for: Dimethylethanolamine Decreases Epileptiform Activity in Acute Human Hippocampal Slices in vitro
Source: Front Mol Neurosci. 2019 Sep 6;12:209. doi: 10.3389/fnmol.2019.00209 (PMC6743366; doi:10.3389/fnmol.2019.00209)
Supplement: TABLE S1 — Clinical data of patients, who provided tissue for electrophysiological assessment of the effects of DMEA on epileptic activity. [file Table_1.docx]

Supplementary Material

**Supplement Table 1. Clinical data of patients, who provided tissue for electrophysiological assessment of the effects of DMEA on epileptic activity.**

| Patient ID | Age | Sex | Onset age | AEDs (mg/day) at time of resection | Seizures / month (mean) | Histology |
| --- | --- | --- | --- | --- | --- | --- |
| 1 | 26 | m | 11 | OXC (2100), CLB (20) | 4.5 | HS Wyler 2 |
| 2 | 52 | f | 33 | LEV (3000), CBZ (1500) | 0.25 | astrogliosis |
| 3 | 43 | m | 29 | LTG (500), LAC (500), CLB (20) | 3 | HS Wyler 1 |
| 4 | 36 | m | 12 | LTG (200), LAC (400) | 3.5 | HS Wyler 2 |
| 5 | 33 | m | 15 | LTG (400) | 1.5 | astrogliosis |
| 6 | 31 | m | 21 | LAC (600), BRV (200) | 12 | mMCD type 1 |
| 7 | 22 | m | 15 | LTG (800), ESL (1200) | 6 | astrogliosis |
| 8 | 40 | m | 26 | CBZ (1200), TPA (250), BRV (200) | 3.5 | FCD type 2a |
| 9 | 27 | m | 10 | LAC (400), BRV (50) | 4.5 | HS Wyler 2 |
| 10 | 22 | m | 19 | LTG (300), LAC (400) | 1 | HS Wyler 1 |
| 11 | 34 | m | 6 | LTG (700) | 1.5 | HS Wyler 1 |
| 12 | 21 | f | 17 | OXC (1000) | 5.5 | HS Wyler 3 |

Abbreviations: BRV, brivaracetam; CBZ, carbamazepine, CLB, clobazam; ESL, eslicarbazepine acetate; FCD, focal cortical dysplasia; HS, hippocampal sclerosis; LAC, lacosamide; LEV, levetiracetam; LTG, lamotrigine; mMCD, mild malformation of cortical development; OXC, oxcarbazepine; TPM, topiramate; VPA, valproate; ZNS, zonisamide
