# Supplementary material for: Dimethylethanolamine Decreases Epileptiform Activity in Acute Human Hippocampal Slices in vitro
Source: Front Mol Neurosci. 2019 Sep 6;12:209. doi: 10.3389/fnmol.2019.00209 (PMC6743366; doi:10.3389/fnmol.2019.00209)
Supplement: TABLE S2 — Summarized results of burst activity in human tissue during application of DMEA, LAC, no intervention or sucrose as control for osmolarity effects. [file Table_2.docx]

Supplementary Material

**Supplementary Table 2. Summarized results of burst activity in human tissue during application of DMEA, LAC, no intervention or sucrose as control for osmolarity effects**

|  | **Amplitude [mV]** | | | **Inter-event interval [s]** | | | **Number of events [n]** | | |
| --- | --- | --- | --- | --- | --- | --- | --- | --- | --- |
|  | **baseline** | **inter-**  **vention** | **wash-**  **out** | **baseline** | **inter-vention** | **wash-**  **out** | **baseline** | **inter-vention** | **wash-out** |
| **5 mM DMEA** | 0.62 ± 0.1 | 0.57 ± 0.1 | 0.62 ± 0.1 | 1.73 ± 1.4 | 2.01 ± 1.0 | 2.17 ± 1.1 | 264.3 ± 142.2 | 178.3 ± 75.0 | 135.1 ± 94.5 |
| **10 mM DMEA** | 0.52 ± 0.2 | 0.42 ± 0.3 | 0.53 ± 0.2 | 2.22 ± 1.4 | 82.1 ± 130.4 | 2.48 ± 1.0 | 212.9 ± 130.8 | 99.57 ± 85.2****** | 153 ± 81.7 |
| **LAC** | 0.47 ± 0.1 | 0.43 ± 0.1 | 0.47 ± 0.1 | 1.43 ± 0.5 | 3.1 ± 0.4***** | 2.47 ± 1.1 | 238.3 ± 110.1 | 88.25 ± 25.2***** | 136.5 ± 55.2 |
| **no intervention** | 0.56 ± 0.4 | 0.51 ± 0.4 | 0.54 ± 0.4 | 1.21 ± 0.3 | 1.95 ± 0.8 | 2.47 ± 1.7 | 257 ± 60.7 | 175 ± 83.8 | 159 ± 91.5 |
| **Sucrose** | 0.38 ± 0.1 | 0.35 ± 0.1 | 0.41 ± 0.2 | 2.24 ± 1.6 | 4.6 ± 4.0 | 3.91 ± 3.2 | 189.2 ± 111.4 | 129.3 ± 87.70 | 157.2 ± 111.3 |

Results are presented as mean ± SD of all recordings for each application phase; 5 mM DMEA (n=6), 10 mM DMEA (n=10), lacosamide (n=4), no intervention (n=3), sucrose (n=3); Asterisks mark significant differences as assessed by One-way ANOVA and post-hoc with Tukey’s multiple comparison test (*p <0.05, **p <0.01 compared to baseline).
